# Thermoresponsive Polymers under Solvent Flow through Molecular Dynamics

**DOI:** 10.1021/acs.jpcb.5c07833

**Published:** 2026-03-13

**Authors:** Scott D. Hopkins, Estela Blaisten-Barojas

**Affiliations:** Center for Simulation and Modeling (formerly, Computational Materials Science Center) and Department of Computational and Data Sciences, 3298 George Mason University, Fairfax, Virginia 22030, United States

## Abstract

Common computational methods for describing laminar flow of dilute polymer solutions (LFDPS) in computational physical chemistry and engineering, such as continuum fluid dynamics approaches for the solvent description in conjunction with coarse-grained modeling for the solvated polymers, rely on sets of user-provided parameters poorly amenable to reproduce specific molecular characteristics at the atomic scale of the addressed system. In recent years, a flow molecular dynamics methodology has been shown to be a viable approach for simulating flows of molecular solutions. However, cases developed so far for condensed phase modeling based on this approach have been highly scarce. Here, we investigate the suitability of a de novo nonequilibrium molecular dynamics NEMD as adapted through our custom modified OPLS-AA force field and applied to LFDPS considering three solvents of different viscosities, water, a 50:50 water/glycerol mixture, and glycerol, and two thermoresponsive polymer derivatives of polyacrylamide, PNIPAM and PDEA. We show that the strengths of both computational approaches yield a descriptive atomistic perspective of the directed flow applied to dilute low molecular weight (LMW) polymer solutions in all of the three solvents considered, evidencing along 200 ns the spatiotemporal mechanism of energy and polymer structure changes that an applied flow triggers for elongating a globular polymer without modifying the laminar behavior of the flowing solution. We additionally demonstrate that the mechanism for the polymer structure change from globular to extended coil requires that the applied flow velocity should be at or above a threshold value *v*_th_ for polymer elongation to occur, thus evidencing a novel simulation parameter. By employing two LMW polymers that are thermoresponsive, easy to synthesize, and commercially reachable, we further demonstrate with pioneering in silico experiments that the LFDPS systems are realizable at temperatures 10–40 K higher than standard thermodynamic conditions. Hence, in silico LFDPS experiments with thermoresponsive polymers have two physics-based parameters, the flow velocity of the solution and the temperature variations around the lower critical solution temperature, making them a desirable selection for several applications including microfluidics for analyses and biosensing where the polymer’s ability to stretch and contract is fundamental.

## Graphical abstract

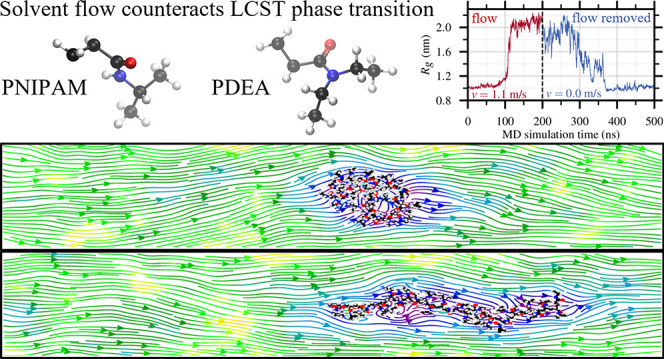

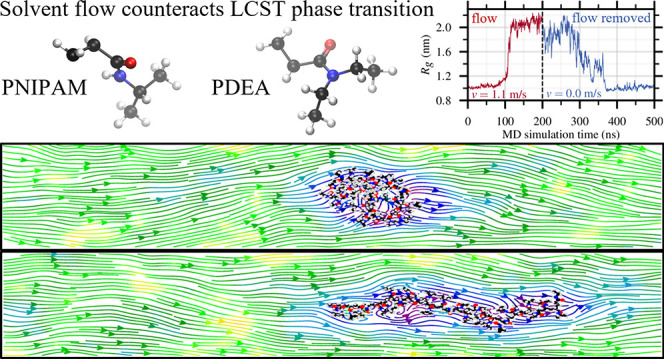

## 1 Introduction

Novel applications have emerged by employing laminar flow of dilute polymer solutions (LFDPS) based on unique polymer properties, such as chain stretching, elasticity, and viscosity increase under shear, which enables the control of flow and fluid transport. Among them, in microfluidics for biosensing and molecular analyses, the polymer elasticity helps profile molecules, while for pipeline drag reduction entailing lower pumping costs, it is the polymer’s ability to stretch and contract that acts as shock absorbers. Meanwhile, for oil recovery with an improved sweep efficiency, the enhancement is due to the injected water viscosity increase enabled by polymer addition. The interdisciplinary field of microfluidics aims at controlling small volumes of flowing liquids in the range of 10^–3^ to 10^–6^ liters or less circulating in microchannels for applications in chemical synthesis,^1^ lab-on-a-chip devices,^2^ drug delivery,^3^ among others.^4^ The fluid flow in these channels is typically laminar. While the fluids employed for bioapplications are primarily aqueous solutions, the utilization of glycerol solutions leverages its hygroscopic nature and high viscosity for flow control, as a stabilizing medium in lab-on-a-chip devices, and as a viscosity calibrator. Depending upon the application, it may be advantageous to produce liquid–solute mixing along the channels.^5^ It has been shown in laboratory experiments that under initially laminar flow conditions, a dilute polymer solution may produce elastic turbulence in viscoelastic fluids.^6,7^ These authors investigated the flow quality along microchannels of a viscoelastic fluid containing a low concentration of high *M*_w_ polyacrylamide (PAM) that is weakly thermoresponsive. They observed that at low Reynolds numbers, the fluid flow became unstable downstream, consistent with the characteristics of elastic turbulence. In their experimental setup, initially, the flowing polymers were elongated by flowing around cylindrical obstacles, and then traveling downstream, they were able to decrease their length due to structural fluctuations and the flow velocity differential. Fluctuations of the polymer length were conceptually linked to an elastic turbulent flow sustained by sudden stretching–elongation events. A recent computational fluid dynamics article^8^ corroborated that vortices can be produced in viscoelastic fluids if there is a spatiotemporal energy localization in the neighborhood of flowing polymers modeled by the finite extensible nonlinear elastic (FENE-P) dumbbells.^9,10^

In this paper, our aim is the atomistic exploration of the spatiotemporal localization of energetic and structural changes that may occur in LFDPS. Our all-atom implementation of a directed flow applied to a dilute polymer solution is a pioneer in the field. We selected six solutions containing three solvents of different viscosities and two solutes that are thermoresponsive linear polymers.^11^ In recent years, multiple uses of synthetic low molecular weight (LMW) poly­(*N*-isopropylacrylamide) (PNIPAM) have emerged, such as drug delivery,^12,13^ due to the sharp coil–globule phase transition that occurs at near human body temperature.^14,15^ Solvated PNIPAM has an extended conformation due to solvent swelling. However, this solvated extended polymer has the ability of deswelling and become a compact globular structure in response to slight temperature increases near the lower critical solution temperature (LCST).^16^ In fact, the swelling/deswelling mechanism is thermoreversible, enabling the same polymer chain in the solvent to become extended–compacted–extended–compacted many times with small temperature changes around its LCST. Another polymer that has a sharp LCST in the range of 300–310 K in water is poly­(*N*,*N*-diethylacrylamide (PDEA),^17^ which has been used as a trigger to form thermoreversible gels,^18–20^ along with other healthcare materials. Both PNIPAM and PDEA are derivatives of PAM that share the same backbone and have PAM’s hydrophilic amide side groups augmented by *N*-isopropyl in PNIPAM and by *N*,*N*-diethyl in PDEA, introducing a hydrophobic component to the side chains of the two polymers.

In this paper, we address the first of its kind in silico experiment of LFDPS at the nanoscale scale through a de novo nonequilibrium molecular dynamics (NEMD) approach that enables us to follow in time the atomistic characteristics of a flowing dilute LMW polymer solution. We investigated the behavior of LMW polymer chains of PNIPAM and PDEA under a directed flow when solvated in water, glycerol, and a 50:50 glycerol/water mixed solvent by first determining that a threshold flow velocity *v*_th_ is required for triggering the solvated polymer structural transition between the globule and extended configurations at the LCST and above for each solution considered. This in silico exploration relies heavily on the availability of high-performance computing platforms for its execution. The methodologies involved and the molecularly developed polymer and solvent models are extensible for treating LFDPS with polymers of any *M*_w_ as long as the system remains at the nanoscale reliable reach of NEMD. The article manuscript is organized as follows: Section 2, Models and Methodology, details the tools and procedures used to simulate the systems and solvent flows with the custom modified, all-atom nonequilibrium molecular dynamics simulations. Section 3, Results and Discussion, provides simulation analyses, interpretation, and discussions centered on the simulation observations focused around polymer–solvent interaction energies, polymer structural properties, solution temperature, and flow velocity. Section 4, Conclusion, summarizes observations and discoveries. Additional quantitative details are provided in the Supporting Information (Supporting Information).

## 2 Models and Methods

PNIPAM and PDEA polymer chains containing 30 monomers were modeled with syndiotactic tacticity establishing an alternating orientation of the side groups about the polymer backbone. Through a de novo all-atom nonequilibrium molecular dynamics approach, this article advocates for a unified narrative of the pioneer in silico experiment of laminar flow of dilute polymer solutions at the atomic scale. We have evidenced that two LMW thermoresponsive polymers, 30-PNIPAM and 30-PDEA of *M*_w_ 3400 and 3800 u, solvated in three different liquids at their LCST, undergo a flow-counteracted structural transition from their globule structure at the LCST to an extended coil structure through the application of a directed flow with a characteristic flow velocity termed threshold velocity. The PNIPAM monomer has 19 atoms, and the chemical structure of a polymer chain with 30 monomers is H-[CH_2_CH­(CONHCH­(CH_3_)_2_)]_30_-H containing 572 atoms with *M*_w_ = 3396.819 u. The PDEA monomer has 22 atoms, and the chemical structure of a polymer chain with 30 monomers is H-[CH_2_CH­(CON­(CH_2_CH_3_)_2_)]_30_-H containing 662 atoms with *M*_w_ = 3817.629 u. In both cases, the polymer chain ends are capped with hydrogen atoms. Based on the *M*_w_ of polymer chains, when *M*_w_>10000 u, “polymer behavior” is expected, while “oligomer behavior” characterizes polymer chains with *M*_w_<10000 u because their molecular properties might be slightly affected by removing or adding monomers.^21^ The two polymer chain models considered are in the oligomer regime of polymer *M*_w_ and were selected based on our previous study^22^ determining that for linear acrylic polymers, the globule structure was as compact as could be in 3D for polymer chains with more than 20 repeating units. Therein, the modeled polymer chains are termed 30-PNIPAM and 30-PDEA. A consideration in modeling a liquid solution at the nanoscale is that once the solute polymer is selected, the size of the elongated computational box containing the solvent molecules is commensurate with the polymer chain extended length. The solvents considered were water, glycerol, and a 50:50 water/glycerol mixture.

The SPC/E model^23^ was used for water, while the two polymers and the glycerol molecules were modeled with the all-atom OPLS/AA force field^24^ modified by custom restrained electrostatic potential (RESP)^25^ partial atomic charges. Notably, custom-based force fields such as ours for synthetic polymers are paramount.^26^ Initial configurations of the polymer chains in typical coil and globule structures were taken from a previous publication.^27^ Figure 1 depicts an instantaneous structure of each polymer chain with a blowup detailing the monomer atomic structure. Similarly to our previous approach for modifying the all-atom force field,^22^ the partial atomic charges were calculated for the full 30-PNIPAM and 30-PDEA molecules in the globule structure at the density functional theory B3LYP/6-31G­(d) level,^28,29^ with GD3 dispersion correction^30^ and the Merz–Singh–Kollman population analysis,^31^ as implemented in Gaussian 16.^32^ The electronic energy of 30-PNIPAM was −10960.107 Ha for the globule and 265.326 kJ/mol higher for the coil configuration, while for 30-PDEA, the globule electronic energy was −12139.110 Ha, with the coil configuration energy being 162.121 kJ/mol higher. Hence, the globular structure of PNIPAM requires significantly higher energy to destabilize into the coil structure than does the PDEA globule. Topology files of the two polymer chains globular structures are available,^33^ and the Supporting Information includes pertinent details.

**Figure 1:**
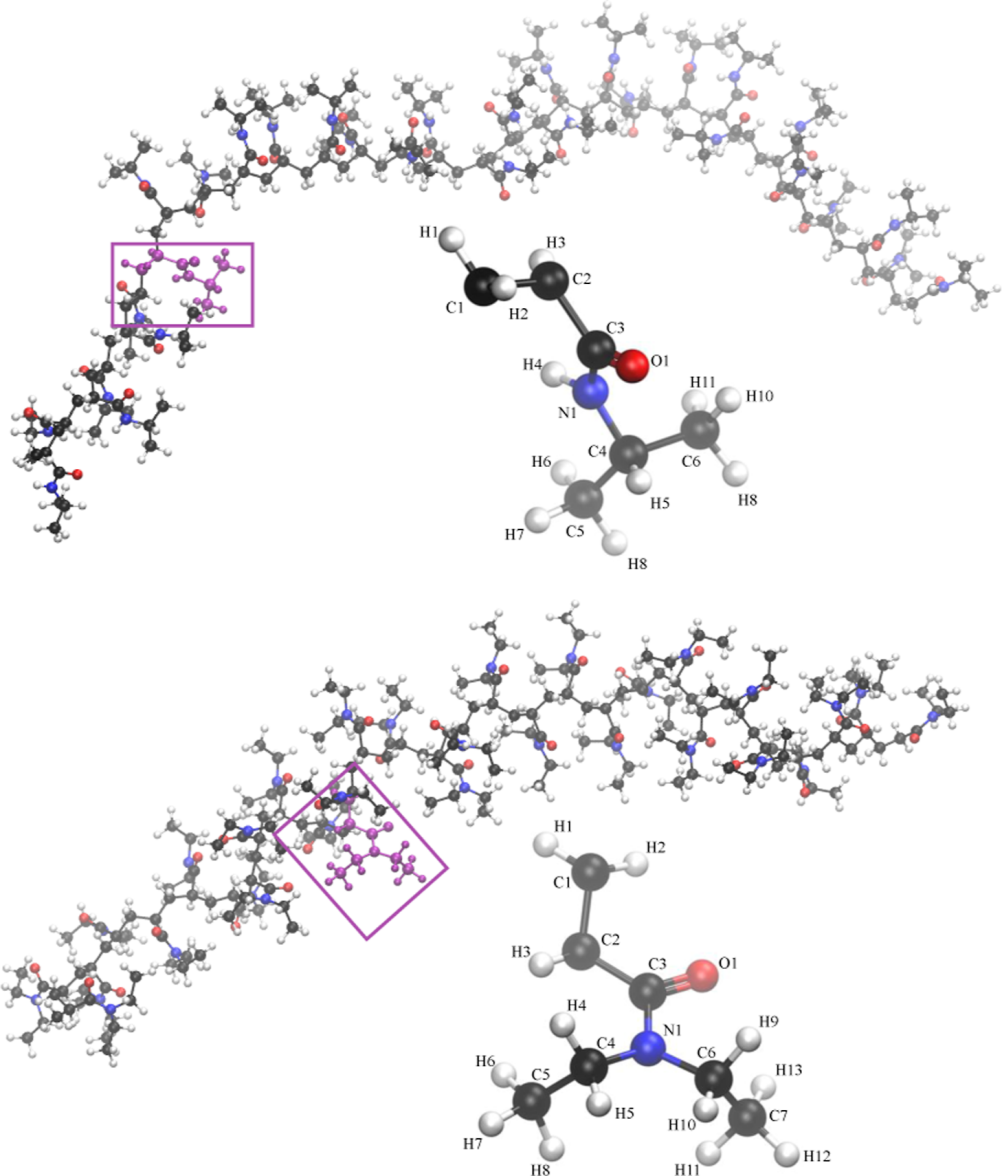
Chemical structure of the syndiotactic 30-PNIPAM (top) and 30-PDEA (bottom) along with their monomer subunits and imaged by atom type (C = black, O = red, N = blue, H = white). Head and tail monomers have an additional H atom required to cap the polymer chain.

The NEMD-AA simulations were performed using GROMACS (version 2018.8).^34^ The globule structure of 30-PNIPAM and 30-PDEA was first simulated in vacuum at LCST + 20 K to ensure stability of the compact globular structure of the two thermoresponsive polymers. Each final globular configuration of either 30-PNIPAM or 30-PDEA was then placed in a 20 nm × 5 nm x 5 nm computational box and solvated with either water, glycerol, or a 50:50 w/w glycerol/water mixture, as depicted in Figure 2. The resulting system contained approximately 16110 water molecules, 8260:1620 water/glycerol molecules, and 3420 glycerol molecules, yielding a 30-PNIPAM relative concentration (*C*_poly_) of 1.15, 1.13, and 1.06 (% w) and a 30-PDEA *C*_poly_ of 1.30, 1.27, and 1.19 (% w), respectively. To verify the reproducibility of the simulations, three different system samples with similar initial geometries of the polymer chains and similar number of solvent molecules were performed. A full description of each system sample composition is contained in Tables S1 and S2 of the Supporting Information. The results from the three system samples were basically identical. In what follows, only one of these system samples is described and illustrated.

**Figure 2:**
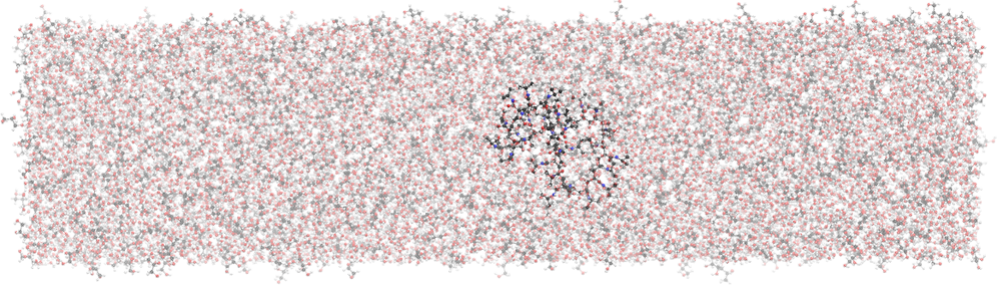
Depiction of the 20 nm × 5 nm × 5 nm computational box with an initial configuration of the globular 30-PNIPAM immersed in the 50:50 w/w glycerol/water liquid mixture at 300 K. The solvent contains 8260:1620 water/glycerol molecules and *C*_poly_ = 1.13.

To equilibrate these systems, the polymer chain in its predetermined globular structure was restrained to a fixed position near the center of the elongated computational box, while the full system (polymer chain plus liquid solvent) was NPT-MD equilibrated at the LCST and 101.325 kPa along a 40 ns simulation run. The Nosé–Hoover thermostat^35,36^ with a 1.0 ps time constant and the Parrinello–Rahman isotropic barostat^37^ with a 2.0 ps time constant were used. For each polymer–solvent combination, two temperatures were considered: the LCST and LCST + 20 K. For 30-PNIPAM, the LCST is around 300 K in water, 320 K in the 50:50 glycerol/water mixture, and 380 K in glycerol. For 30-PDEA, the LCST is around 300 K in water, 340 K in 50:50 glycerol/water, and 390 K in glycerol.^27^ Atoms in the polymer chain were restrained during equilibration with a harmonic force constant of 1000 kJ/mol/nm^2^. The linear constraint solver (LINCS)^38^ was applied to all bonded hydrogens in the solvent and the polymers. A 1.0 fs time step, 1.2 nm van der Waals and electrostatic cutoffs, and periodic boundary conditions were employed. The smooth particle-mesh Ewald method^39^ was implemented with a Fourier spacing of 0.12 nm to handle long-range electrostatic interactions.

The solvent directed flow was simulated through a de novo NEMD modification based on the pull-force routine^40,41^ while keeping the NVT-MD system conditions. This modified pull-force routine works by applying a directional force that pulls each solvent molecule present within a defined region of the computational box along its elongated direction. Hence, in our systems, the pull-force was present for any molecule situated in the computational box between *x* = 0 and *x* = 8 nm. The applied forces accelerate the solvent molecules within that specific region to a terminal flow velocity, while the resulting flow velocity in the remainder of the box depends on the applied force strength characterized by a force constant value; higher force constants result in higher molecular velocities. To verify the method, a system containing only solvent molecules and no polymer was created, which resulted in a laminar flow. Downstream, this methodology introduces a slight gradient in the flowing solution density. Figure S1 gives an illustration of this effect. After 40 ns of equilibration for systems without flow, the production runs of the flowing solution containing the immersed polymer were performed over 200 ns with a time step of 2.0 fs, while reported results are averaged over the final 30 ns. To prevent the polymer from moving downstream, the polymer chain’s first backbone carbon atom (C1) of the head monomer was held fixed at the computational box point (10 nm, 2.5 nm, 2.5 nm). To evaluate the flow velocity, a representation of the system velocity field was obtained by dividing the computational box volume into a grid of 4000 cubic cells of 0.5 nm edge size. The position of any solvent molecule within each grid cell was tracked over time to obtain an average solvent velocity per cell across the full grid. This velocity field representation can be inspected visually and numerically.

The liquid system is characterized by Reynolds number Re = (ρ*uL*)/η, where ρ is the solvent density, *u* is the solvent velocity, *L* is a characteristic dimension, and η is the dynamic viscosity. For our system, *L* = 20 nm (length of the computational box), ρ is the equilibrated density of one 30-PNIPAM or one 30-PDEA solvated in each liquid considered, water, glycerol, and their 50:50 mixture. The dynamic viscosity is solvent- and temperature-dependent, ranging from 5.79 × 10^–4^ N-s/m^2^ in water at 320 K^42^ to 1.19 × 10^–2^ N-s/m^2^ in glycerol at 380 K.^43^ If Re is less than 2300, the flow is considered laminar, while if Re is greater than 2900, the flow is considered turbulent. In our simulations, the flow was laminar. From Stokes’ law, the applied drag force *F*_d_ on an object moving in a fluid at very small Re is *F*_d_ = 6πη*R*_g_ *v*, with *R*_g_ and *v* being the object radius of gyration and velocity, respectively.

The interaction energy between the solvent and polymer chain, *E*_int_, was evaluated along all simulations. This interaction includes the electrostatic interaction given by the Coulomb potential and the 12–6 Lennard-Jones potential representing the dispersion term between any two nonbonded atoms at distances below the cutoff of 1.2 nm. The calculation of *E*_int_ was obtained from

1 $$E_{\mathrm{int}}=E_{total}-E_{solvent}-E_{polymer}$$

where *E*_total_ is the total potential energy of the system composed of the liquid within the computational box and one immersed polymer chain, while *E*_solvent_ and *E*_polymer_ are the potential energies of the isolated liquid solvent and the isolated polymer chain, respectively.

## 3 Results and Discussion

Multiple simulations of the applied directed flow to each of the six solutions investigated (three solvents containing either 30-PNIPAM or 30-PDEA) were carried out for determining the flow velocity threshold *v*_th_ at the LCST, which remained the same up to LCST + 20 K. Knowing the *v*_th_ at a desired solution temperature is crucial for our analysis. Indeed, several averaged structural properties of the polymer chain in each of these solutions at the LCST and subjected to a directed flow with its specific flow velocity *v*_th_ were calculated, including radius of gyration *R*_g_, end-to-end distance *R*_ee_, solvent accessible surface area SASA, as well as averages of the polymer–solvent interaction energy *E*_int_ and the drag force *F*_d_. Property averages are given in Table 1. Average values of the 30-PNIPAM or 30-PDEA globule and coil structures, termed phase, were collected along the 200 ns simulation span of the solution under the directed flow at the *v*_th_ of each solution and averaged during the initial 30 ns for the globule and along the final 30 ns for the extended coil.

**Table 1:** NEMD Simulation Summary for Systems at the LCST for Each Flowing Solution Including the Threshold Flow Velocity *v*_th_, the Polymer Chain Radius of Gyration *R*_g_, SASA, End-to-End Distance *R*_ee_, the Polymer–Solvent Interaction Energy *E*_int_/Monomer, and the Drag Force *F*_d_

oligomer	solvent	LCST (K)	*v*_th_ (m/s)	phase	*R*_g_ (nm)	SASA (nm^2^)	*R*_ee_ (nm)	*E*_int_ (kJ/mol)	*F*_d_ (pN)
PNIPAM	water	300	1.1 ± 0.3	globule	1.00 ± 0.02	32.6 ± 0.8	1.00 ± 0.07	–81 ± 3	16.0 ± 0.1
				extended	2.01 ± 0.09	41.7 ± 0.8	6.28 ± 0.49	–94 ± 3	32.2 ± 0.3
	50:50	320	0.4 ± 0.2	globule	1.03 ± 0.03	33.4 ± 1.1	1.05 ± 0.14	–78 ± 3	22.0 ± 0.4
				extended	2.12 ± 0.05	41.7 ± 0.8	6.72 ± 0.26	–92 ± 3	45.2 ± 0.5
	glycerol	380	0.2 ± 0.2	globule	1.10 ± 0.06	34.8 ± 1.9	1.08 ± 0.12	–75 ± 4	54.5 ± 2.8
				extended	2.09 ± 0.04	41.4 ± 0.7	6.83 ± 0.16	–90 ± 3	103.1 ± 1.7
PDEA	water	300	0.4 ± 0.2	globule	1.03 ± 0.02	34.8 ± 0.9	1.44 ± 0.28	–71 ± 2	7.3 ± 0.1
				extended	1.84 ± 0.04	41.2 ± 0.7	6.01 ± 0.21	–78 ± 2	13.0 ± 0.1
	50:50	340	0.3 ± 0.2	globule	1.04 ± 0.02	34.1 ± 0.8	1.03 ± 0.15	–72 ± 2	16.6 ± 0.2
				extended	1.77 ± 0.06	40.4 ± 0.7	5.62 ± 0.41	–79 ± 2	28.3 ± 0.6
	glycerol	390	0.2 ± 0.2	globule	1.17 ± 0.05	37.3 ± 1.0	1.06 ± 0.16	–64 ± 3	57.6 ± 2.3
				extended	1.88 ± 0.03	41.4 ± 0.7	5.90 ± 0.17	–75 ± 2	92.7 ± 1.7

The information in Table 1 leads to a detailed atomistic description of the process that the polymer chains in solution undergo when the directed flow at *v*_th_ is applied to the previously equilibrated solution at or above the LCST. In fact, in the span of 200 ns, each polymer chain in its native globular structure swells and elongates into an extended coil conformation. It is noted that the *R*_g_ of the extended coil polymer conformation acquired in the flowing solvent is approximately 15% larger than the *R*_g_ of a typical coil conformation in a nonflowing solvent below the LCST.^27^ For 30-PNIPAM in the three considered solvents, the flow-induced globule-to-extended coil structure change is evidenced by the *R*_g_ doubling in size, the *R*_ee_ turning six times as long, and the SASA increasing by about 50%. These macromolecular structural changes lead to a doubling of the drag force exerted on the polymer. Additionally, for these structural changes to occur, the polymer–solvent interaction energy *E*_int_/monomer turns more negative by about 10 kJ/mol, consistent with the hydrophilic propensity improvement of the swelled polymer. Hand-in hand, in the extended coil structure, the polymer backbone bonding terms remained unchanged, while distances between the polymer side groups were longer, yielding a net increase of the intrapolymer electrostatic and dispersion energies. We assert that this localized energetic mechanism entails a decrease in the kinetic energy of the solvent molecules located in the polymer vicinity, as pictorially shown in Figures 3 and 4. In previous work,^27^ we established that without flow in the same three solutions, the 30-PINIPAM coil-to-globule phase transition was triggered when the solution temperature was increased to the LCST and occurred due to electrostatic and dispersion components of *E*_int_ becoming less negative, while the backbone bonding terms remained unchanged with twists to accommodate the side groups minimizing the distances between them. We also determined that only a negligible number of transient polymer–solvent hydrogen bonds were formed or annihilated. For 30-PDEA the structural changes from globule-to-extended coil due to the applied directed flow at its *v*_th_ are less steep than in 30-PNIPAM, with an *E*_int_/monomer decrease of about 8 kJ/mol. The latter is lower than that in 30-PNIPAM, pointing to a more modest swelling. However, the mechanism of the process triggered by the applied flow is the same in both polymer chains. Hence, our battery of NEMD-AA simulations is a quantitative, atomistic verification of the localized spatiotemporal mechanism of energy exchanges the flow imposes on the solution for changing the native globular structure of the polymer chain at the LCST into an extended coil structure at that temperature.

**Figure 3:**
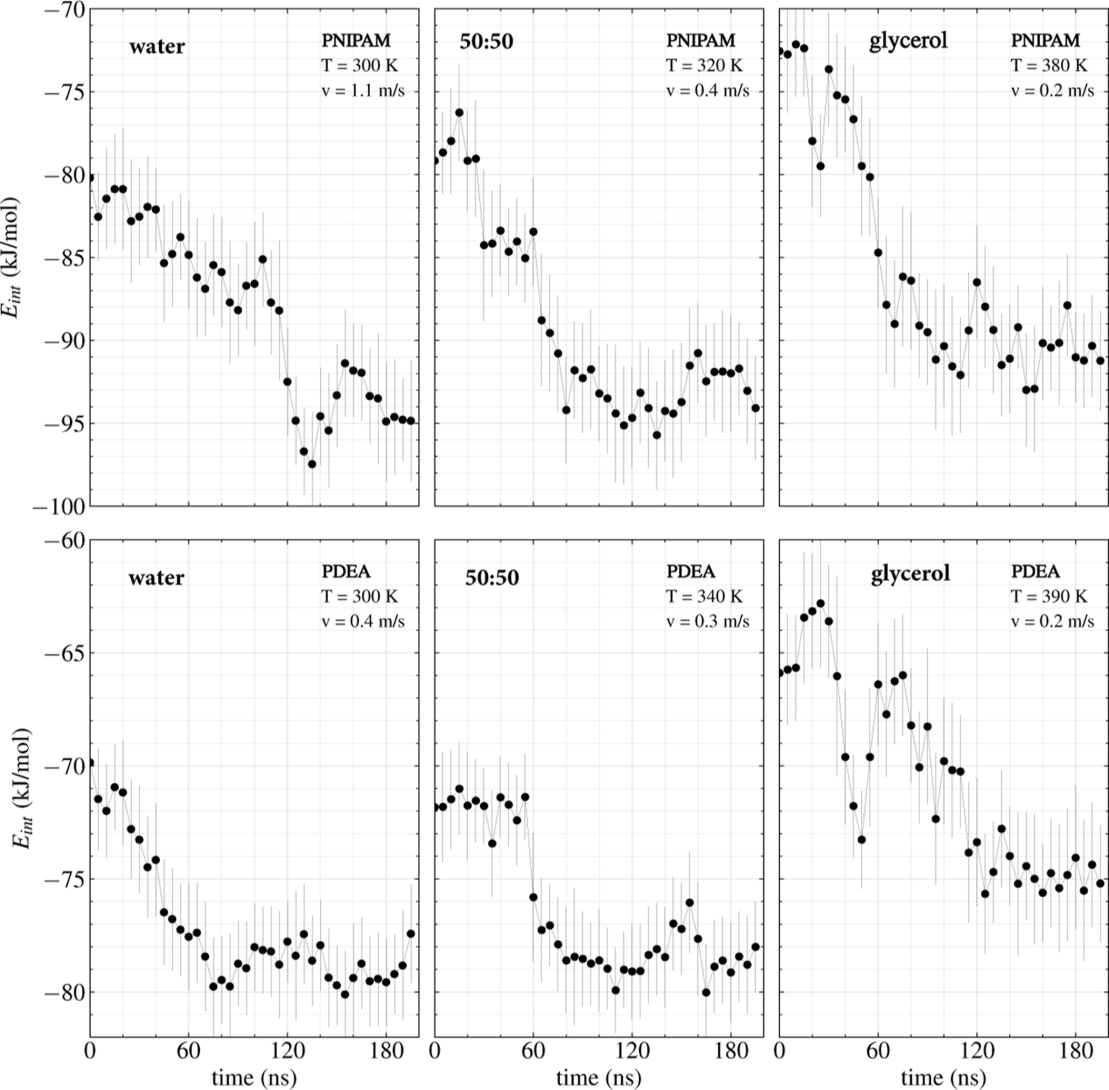
Polymer–solvent interaction energy *E*_int_/monomer in the flowing 30-PNIPAM (top) and 30-PDEA (bottom) at the LCST and the threshold flow velocity *v*_th_ in water, 50:50 w/w water/glycerol, and glycerol. Points correspond to averages over 5 ns of simulation time and the corresponding standard deviations are shown as error bars.

**Figure 4:**
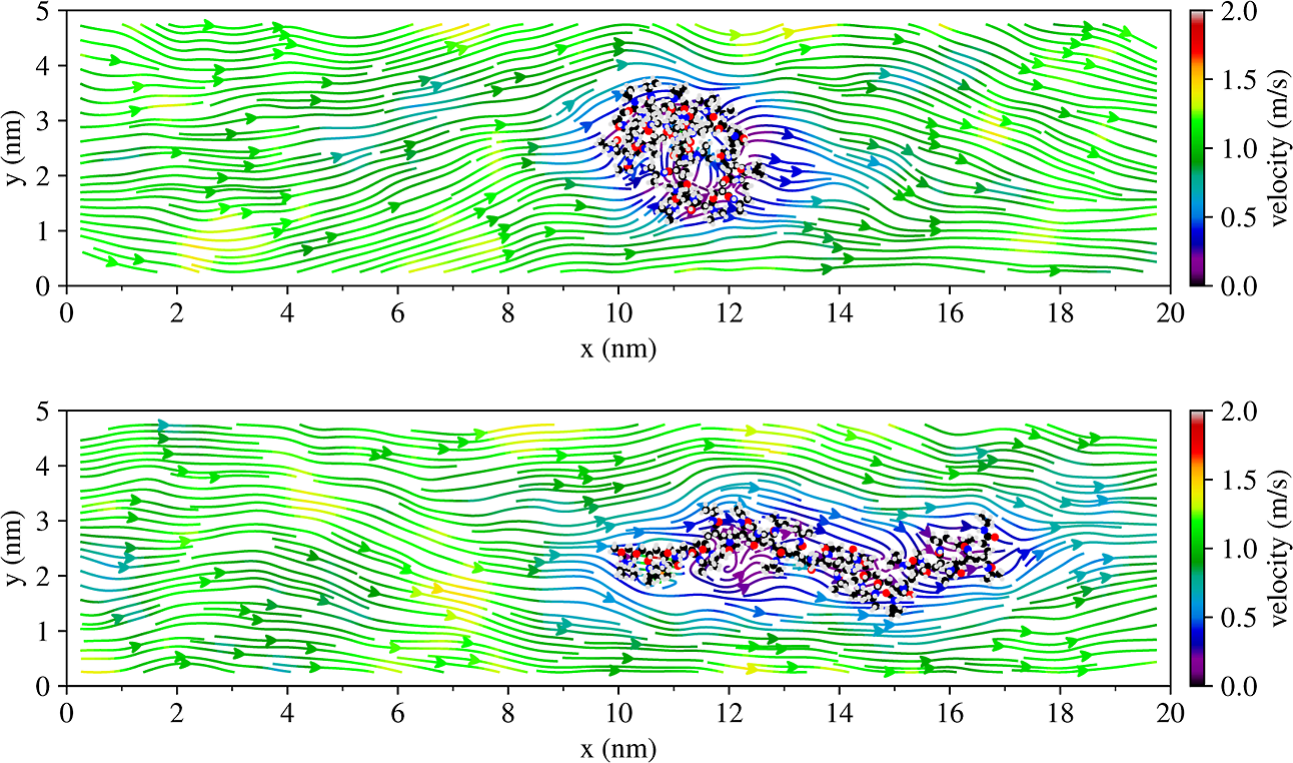
Flow field of 30-PNIPAM in water system at the threshold velocity of 1.1 ± 0.3 m/s, with the polymer in both the globule (top) and extended (bottom) configurations at 300 K. Atoms in 30-PNIPAM are colored as C = black, O = red, N = blue, and H = gray.

Furthermore, the polymer chain elongation process is not instantaneous and requires between 50 and 100 ns to start the nanoscale extension mechanism. Figure 3 depicts the time evolution of the polymer–solvent interaction energy, *E*_int_/monomer, for 30-PNIPAM and 30-PDEA flowing in each of the three solvents studied at their corresponding system LCST and *v*_th_. For example, a threshold flow velocity of *v*_th_ = 1.1 ± 0.3 m/s was required for transitioning the 30-PNIPAM globular structure into an extended coil configuration in water, the process being gradual and settling on the final value around 120 ns of evolution. Meanwhile, *v*_th_ decreased with the addition of glycerol molecules to the solvent, equaling 0.4 ± 0.2 m/s in the 50:50 w/w glycerol/water mixture and 0.2 ± 0.2 m/s in pure glycerol, while a stabilized elongation of the polymer chain started to occur earlier at 90 and 60 ns, respectively. The reason for the faster process in the glycerol-containing solvents is a reduced number of water molecules in the polymer swelling and less hydrophilic propensity. Meanwhile, 30-PDEA in water required a significantly lower threshold flow velocity of *v*_th_ = 0.4 ± 0.2 m/s, maintaining comparable values to the 30-PNIPAM case of 0.3 ± 0.2 m/s in 50:50 w/w glycerol/water and 0.2 ± 0.2 m/s in glycerol. Consistently, the polymer chain elongation process started early at about 60 ns of evolution for the solvents with water. This temporal flag suggests that in water the 30-PDEA is more susceptible to the directed flow action than 30-PNIPAM. The early elongation process start in 30-PDEA relates to the total energy difference between its globule and coil structures, which is about 40% smaller than the equivalent of 30-PNIPAM (see Section 2). For the six considered solutions, the *v*_th_ remained unchanged for solution temperatures up to 20 K above the LCST. The polymers *R*_g_, *R*_ee_, SASA, and applied drag force *F*_d_ reached steady values along the last 30 ns of their overall 200 ns evolution as shown in Figures S2 through S5 for flow velocities at and below the respective *v*_th_.

A visual validation of the in silico experimental discussion of previous paragraphs is pertinent. The flow velocity field was illustrated by slicing the computational box along its elongated direction. The flow velocity field along a slice containing the computational box center is shown in Figure 4 for the 30-PNIPAM in a water system. A more detailed view containing all of the box slices is depicted in Figure 5. The color scale in these figures corresponds to the magnitude of the velocity. In regions where no polymer is present, the flow field is smooth. While there are small variations of higher and lower velocities, in general, the velocity is uniform throughout the system. However, in proximity to 30-PNIPAM, the velocity field can be seen traversing around the molecular structure with a local decrease in the solvent velocity within the polymer chain’s hydrodynamic radius region. The latter effect is present while the polymer chain is globular, as well as after having transitioned into an extended coil conformation.

**Figure 5:**
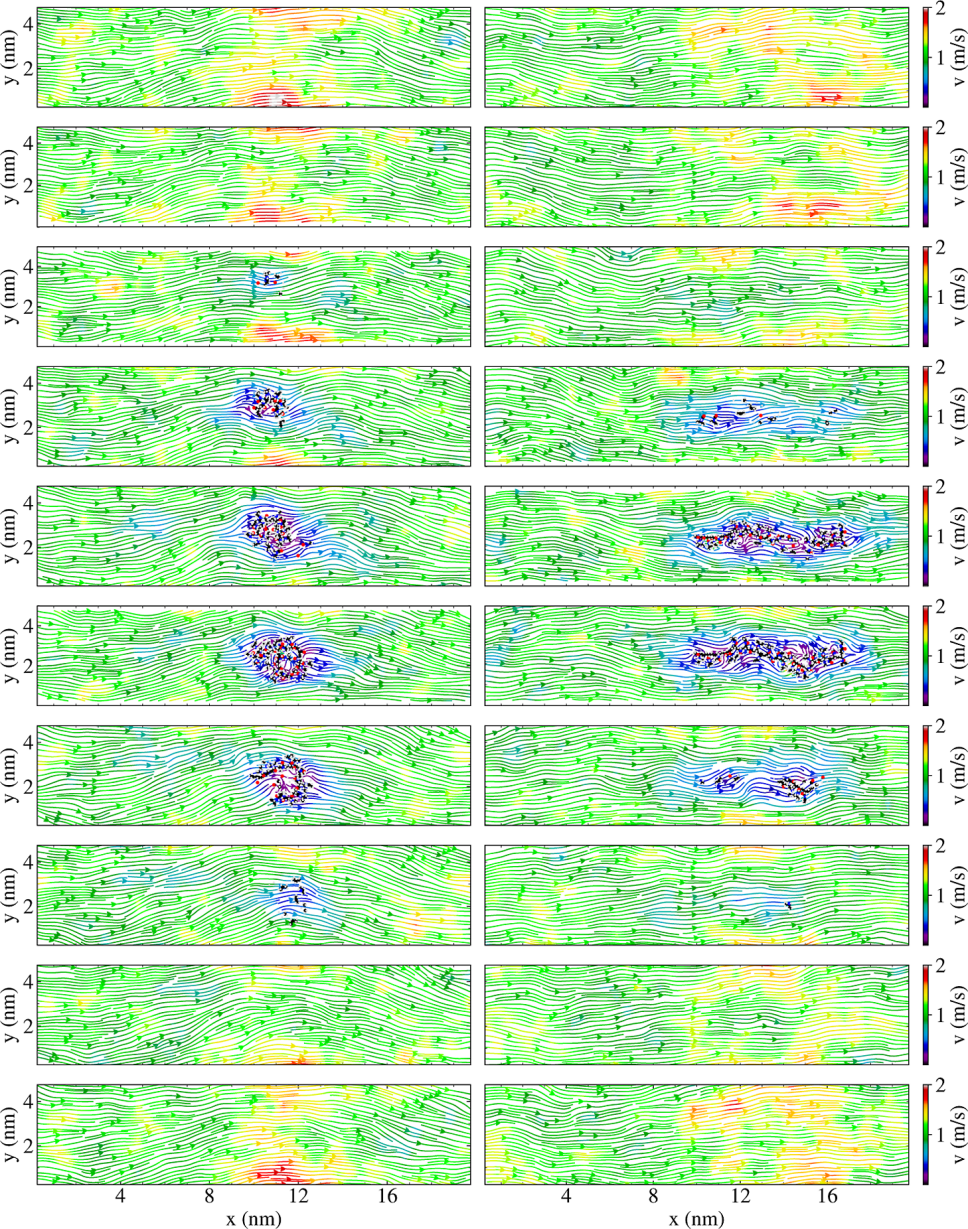
Flow field slices through the *x*–*y* plane for 30-PNIPAM in water at the LCST (300 K). Each slice is approximately 20 nm × 5 nm × 0.5 nm, beginning at slice = 0.0–0.5 nm (top) and ending at slice = 9.5–10.0 nm (bottom). The globule configuration is depicted on the left side of the figure, while the extended coil is on the right. The flow velocity was 1.1 m/s.

To further verify that the polymer extension was the result of the directed flow, additional simulations were performed with the flow velocity turned off, and the system was followed for an additional 300 ns. Once the flow velocity was removed, the polymer chain extended structure very quickly relaxed first into a more typical coiled molecular structure,^27^ which can actually vary in an end-to-end distance by a factor of 2 to 3, and afterward returned to the globular structure. In fact, for the water system at the LCST of 300 K, the polymer transitioned back into a globular configuration within 200 ns and remained in that stable configuration for the remainder of the simulation. Reversibility of the polymer structure by switching on-and-off the applied flow is not a trivial mechanism, which is evidenced in our discourse because of the atomistic modeling approach. For 30-PNIPAM in water at the LCST, Figure 6 depicts the structure reversibility that occurs as time progresses from the extended coil when flow is applied to a globule once the flow is removed.

**Figure 6:**
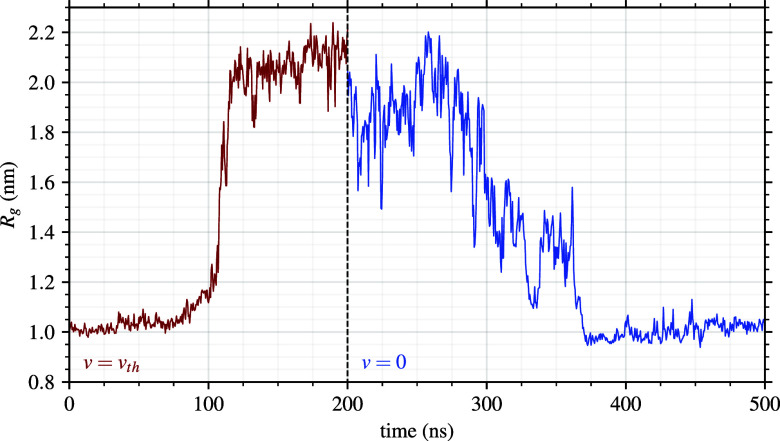
Reversibility of the 30-PNIPAM extended-coil to globule structures in water at 300 K. The polymer in directed flow (left) and when the flow is eliminated (right). The flow velocity *v*_th_ was 1.1 m/s.

For completeness, the radial distribution function (RDF) between the center of mass of each monomer in 30-PNIPAM or 30-PDEA and the center of mass of each solvent molecule was calculated for the globule and extended coil structures in each of the three flowing solvents considered. For the solute globular structure, the RDF was calculated along the beginning 30 ns of each of the 200 ns simulations, while the corresponding calculation for the extended coil structure occurred along the last 30 ns of each simulation at the LCST. These functions are displayed in Figure S6. Because the polymer solutions are flowing, the population of solvent molecules neighboring the polymer is not constant in time, resulting in smearing and blurring of the RDF concept of structure characterized by shells of neighbors. Overall, the RDF peak heights of the extended coil polymer chain are higher than those obtained from its globular structure, validating the statement of the increased hydrophilic behavior of the polymer chain extended coil as compared with the globular case. The 30-PNIPAM in water has a double peak at around 0.4 nm, accounting for water molecules approaching the polymer chain from its end monomers or from the other backbone monomers, while a third peak at around 0.6 nm is visible. In pure glycerol, the peaks occur at 0.55, 0.7, and 1.1 nm. In the 50:50 water/glycerol mixture, both trends are observed for the individual liquid components. The 30-PDEA in water displays two very broad peaks resulting from its wide side groups that limit the number of solvent molecules reaching closer to the backbone. This smearing effect worsens in the glycerol solution, where the first/second peaks at around 0.7 nm appear as merged due to both the large size of the polymer side groups and the increased size of the solvent molecules.

To further validate the statistics behind the data collected for the flowing solution at the *v*_th_, scatterplots between the instantaneous polymer–solvent interaction energy (*E*_int_/monomer) and the polymer radius of gyration *R*_g_ along a simulation are depicted in Figure 7. These data distributions clearly identify the sharp size change of the polymer between the two structures, initially a globule at the LCST, counter-transitioning to the extended coil due to the flow. For 30-PNIPAM in water at 300 K, the globular structure displays tiny size fluctuations even though the spread of its interaction energy per monomer with the flowing solvent is significant, about 25 kJ/mol along 30 ns of evolution. Once the flow elongates the polymer, counter-transitioning the globule to the extended coil, the *R*_g_ evidences a floppy extended coiled polymer chain with an extending-contracting coil structure fluctuating around 2 ± 0.2 nm while entailing a spread of 25 kJ/mol per monomer of the flowing solvent–polymer interaction energy. Overall, the extended coil structure displays a 10–13 kJ/mol decrease in the *E*_int_/monomer with respect to the globule value, indicating that the directed flow induces a more hydrophilic polymer. Similar behavior is observed in the other two solvents. Meanwhile, for 30-PDEA in water, the decrease in *E*_int_/monomer between the globule and extended coil is about 5–6 kJ/mol per monomer, and both structures are floppy with a less pronounced change in *R*_g_. This is likely becoming apparent due to the much lower *v*_th_ in the 30-PDEA system compared to that of 30-PNIPAM. A peculiarity of the 30-PDEA in the glycerol system is that the flow induces two globular stages in the globule phase with slightly different *R*_g_ before the elongation to the extended coil, which relates to the *E*_int_ evolution within the first 100 ns of simulation as shown in Figure 3.

**Figure 7:**
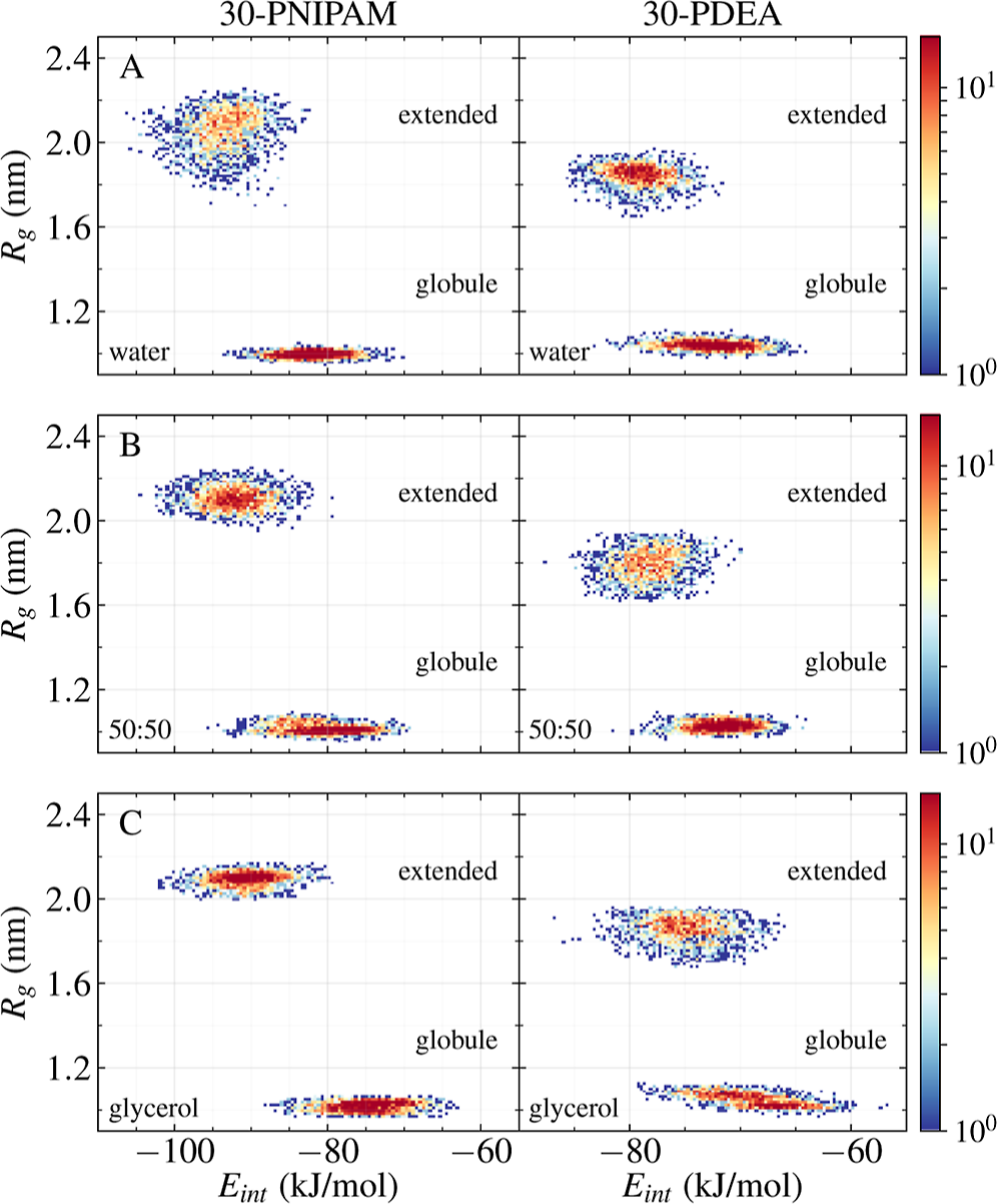
Scatterplots between the solvent–polymer interaction energy *E*_int_/monomer and the 30-PNIPAM (left) and 30-PDEA (right) *R*_g_ at the LCST of each solution along the 0–30 ns time span for the globule structure and along the 170–200 ns time span for the extended coil. (A) In water, (B) in the 50:50 glycerol/water mixture, and (C) in glycerol. The color scale depicts the concentration of a high number of points as red and of low number of points as blue.

In a nutshell, in the laminar flow of dilute LMW polymer solutions of thermoresponsive polymers at or above their LCST, there is an important localized spatiotemporal disturbance at the molecular scale of both the solvent and the immersed polymer chain. A flow-based extension of the polymer generates a substantial increase in its SASA of 7–10 nm^2^, which is accompanied by a lowering of the solvent–polymer interaction energy *E*_int_/monomer of around 8–10 kJ/mol. Additionally, it is corroborated from multiple simulations that an increase of 20 K of the system temperature does not affect the observations at the LCST. For temperatures below the LCST, these polymers are solvated in their flow-generated fluctuating extended coil structure and do not become globular. On the other hand, at the LCST, a reduction of the flow velocity below the *v*_th_ promotes a return of the polymer extended coil to the globule structure. As shown in Figures 4 and 5, when the polymer chain is globular or extended, the solvent flow continues to be uniform within the polymer neighborhood. For polymer chains in the oligomer *M*_w_ regime, we anticipate that the possibility of originating elastic turbulence at the LCST and above will not occur by spontaneous processes of polymer elongation–contraction in solutions with established laminar flow. We conjecture that if the system would have large enough fluctuations of the solution flow velocity around the corresponding *v*_th_, those fluctuations may produce sequences of globule-to-extended coil events, which may generate flow time irregularities of the solution in the proximity of an immersed thermoresponsive polymer chain. We predict that as the polymer *M*_w_ increases beyond 10000 u, for dilute polymer solutions of thermoresponsive polymers at or up to 20 K above the LCST, the polymer elongation mechanism will be affected by longer times for the flow to act on the polymer, without a substantial change in the required *v*_th_ to generate such a process.

## 4 Conclusion

Through a de novo all-atom nonequilibrium molecular dynamics approach, this article advocates for a unified narrative of the pioneer in silico experiment of laminar flow of dilute polymer solutions at the atomic scale. We have evidenced that two LMW thermoresponsive polymers, 30-PNIPAM and 30-PDEA of *M*_w_ 3400 u and 3800 u, solvated in three different liquids at their LCST, undergo a flow-counteracted structural transition from their globule structure at the LCST to an extended coil structure through the application of a directed flow with a characteristic flow velocity termed threshold velocity. The flowing solvents are water, a 50:50 w/w water/glycerol mixture, and glycerol. The LCSTs of 30-PNIPAM in these solvents are 300, 320, and 390 K, while for 30-PDEA, the LCST values are 300, 340, and 380 K for water, a 50:50 w/w water/glycerol mixture, and glycerol, respectively. Typically, the flow counteracted structural transition entails the polymer *R*_g_ doubling in size, the *R*_ee_ elongating by a factor of 6, and the SASA augmenting by about 50%. The process starts as early as 60 ns in the 200 ns simulations and settles in final, fluctuating property values in the subsequent 100–140 ns. A central theme has been the identification of the threshold velocity required to induce this counteracted globule-to-coil structural transition, which via the in silico experiment is determined to be solvent- and polymer-dependent. The water system had the highest threshold velocity, approximately 1.1 ± 0.3 m/s for 30-PNIPAM and 0.4 ± 0.2 m/s for 30-PDEA, while in the 50:50 glycerol/water system, the threshold velocity decreased to approximately 0.4 ± 0.2 m/s for 30-PNIPAM and 0.3 ± 0.2 m/s for 30-PDEA. For the glycerol system, a relatively small threshold velocity of 0.2 ± 0.2 m/s was required to induce the counter-transition of globule into an extended coil configuration for both polymers, due to their modest hydrophilic propensity in this solvent and their lower polymer–solvent interaction energy than in the aqueous solutions. In essence, it was evidenced that an applied flow on the 30-PDEA solutions requires much lower threshold velocities than that on 30-PNIPAM for transitioning their globular structure to an extended coil structure at the LCST and up to 20 K above it.

The behavior of the solvent molecules flowing past the polymer chain and the visualization of the flow at the subnanometer scale were also characterized. The drag force experienced on either polymer chain was calculated, and as expected, it was significantly higher in the viscous glycerol system than in the 50:50 glycerol/water mixture or pure water systems. Additionally, the drag force experienced by each polymer chain at the LCST increased throughout the globule-to-extended coil counteracted transition due to the simultaneous increase of the polymer SASA and hydrodynamic radius. We also verified that if the directed flow was removed in these systems, the flow-extended polymer chain reversed to its native globule structure in about 200 ns at the LCST.

Looking ahead, our spatiotemporal characterization at the atomic scale, evidencing the localization of polymer structural and energetic changes in flowing dilute polymer solutions, may have broad applications in nanoscience and biomedical fields, where precise control over flowing dilute polymer solutions is desired. The in silico spatiotemporal description of the flow effect and possible experimentation at temperatures above thermodynamics normal conditions call for new, high-precision laboratory experiments. Our de novo simulations contribute to the current status of polymer modeling, leading to a richer set of dilute polymer solution applications. Analysis of how flow conditions influence thermoresponsive polymer behavior near human body temperature could inform the design of drug delivery systems or smart biomaterials that respond dynamically to physiological environments.

## Supplementary Material



## Notes

Details on the simulation parameters of the three sample simulations utilized, details of the direct flow effect on the system density, pertinent radial distribution functions along the flowing solutions, and the time evolution along the last 30 ns of the 200 ns simulations of the polymer chain radius of gyration, end-to-end distance, solvent accessible surface area, and the applied drag force for 30-PNIPAM and 30-PDEA polymer chains. The topology files for 30-PNIPAM and 30-PDEA are provided in the Zenodo repository.^33^ (PDF)

## References

[1] Whitesides G. M. (2006). The origins
and the future of microfluidics. Nature.

[2] Stone H. A., Stroock A. D., Ajdari A. (2004). Engineering flows in
small devices:
Microfluidics toward a lab-on-a-chip. Annu.
Rev. Fluid Mech..

[3] Tomeh M. A., Zhao X. (2020). Recent advances in
microfluidics for the preparation of drug and
gene delivery systems. Mol. Pharmaceutics.

[4] Convery N., Gadegaard N. (2019). 30 Years of
microfluidics. J.
Micro Nano Sci. Eng..

[5] Fan, J. ; Li, S. ; Wu, Z. ; Chen, Z. Chapter 3 - Diffusion and mixing in microfluidic devices. In Microfluidics for Pharmaceutical Applications; Santos, H. A. , Liu, D. , Zhang, H. , Eds.; Elsevier, 2018; pp 79–100.

[6] Qin B., Arratia P. E. (2017). Characterizing elastic
turbulence in channel flows
at low Reynolds number. Phys. Rev. Fluids.

[7] Sternberg V. (2021). Elastic turbulence:
An experimental view on inertialess random flow. Annu. Rev. Fluid Mech..

[8] Handler R. A., Blaisten-Barojas E., Ligrani P. M., Dong P., Paige M. (2020). Vortex generation
in a finitely extensible nonlinear elastic Peterlin fluid initially
at rest. Eng. Rep..

[9] Warner Jr. H. R. (1972). Kinetic
theory and rheology of dilute suspensions of finitely extendible dumbbells. Ind. Eng. Chem. Fundam..

[10] Herrchen M., Öttinger H. C. (1997). A detailed
comparison of various FENE dumbbell models. J. Non-Newtonian Fluid Mech..

[11] Gao P., Jiang X., Li J., Nicolas J., Ha-Duong T. (2025). Molecular
simulations of polymer-based drug nanocarriers: From physical and
structural properties to controlled release. Adv. Healthcare Mater..

[12] Qiu Y., Park K. (2001). Environment-sensitive
hydrogels for drug delivery. Adv. Drug Delivery
Rev..

[13] Ward M. A., Georgiou T. K. (2011). Thermoresponsive
polymers for biomedical applications. Polymers.

[14] Dinarvand R., D’Emanuele A. (1995). The use of thermoresponsive hydrogels
for on-off release
of molecules. J. Contr. Release.

[15] Pasparakis G., Tsitsilianis C. (2020). LCST polymers: Termoresponsive nanostructured
assemblies
towards bioapplications. Polymer.

[16] Halperin A., Kröger M., Winnik F. M. (2015). Poly­(N-isopropylacrylamide)
phase
diagrams: fifty years of research. Angew. Chem.
Int. Ed..

[17] Idziak I., Avoce D., Lessard D., Gravel D., Zhu X. X. (1999). Thermosensitivity
of aqueous solutions of poly­(N,N-diethylacrylamide). Macromolecules.

[18] Haddow P.
J., da Silva M. A., Kaldybekov D. B., Dreiss C. A., Hoffman E., Hutter V., Khutoryanskiy V. V., Kirton S. B., Mahmoudi N., McAuley W. J. (2022). Polymer architecture effects on poly­(N,N-diethyl
acrylamide)-b-poly­(ethylene glycol)-b-poly­(N,N-diethyl acrylamide)
thermoreversible gels and their evaluation as a healthcare material. Macromol. Biosci..

[19] Havanur S., Farheenand V., JagadeeshBabu P. (2019). Synthesis and optimization of poly­(N,N-diethylacrylamide)
hydrogel and evaluation of its anticancer drug doxorubicin’s
release behavior. Iran. Polym. J..

[20] Hanyková L., Krakovsky I., Šestáková E., Št’astná J., Labuta J. (2020). Poly­(N,N’,-diethylacrylamide)-based
thermoresponsive hydrogels with double network structure. Polymers.

[21] Naka, K. Monomers, oligomers, polymers, and macromolecules (overview). In Encyclopedia of Polymeric Nanomaterials; Kobayashi, S. , Mullen, K. , Eds.; Springer: Heidelberg, 2017.

[22] Hopkins S. D., Gogovi G. K., Weisel E., Handler R. A., Blaisten-Barojas E. (2020). Polyacrylamide
in glycerol solutions from an atomistic perspective of the energetics,
structure, and dynamics. AIP Adv..

[23] Berendsen H., Grigera J., Straatsma T. (1987). The missing term in effective pair
potentials. J. Phys. Chem..

[24] Robertson M. J., Tirado-Rives J., Jorgensen W. L. (2015). Improved peptide and protein torsional
energetics with the OPLS-AA force field. J.
Chem. Theory Comput..

[25] Bayly C. I., Cieplak P., Cornell W., Kollman P. A. (1993). A well-behaved electrostatic
potential based method using charge restraints for deriving atomic
charges: the RESP model. J. Phys. Chem..

[26] Turney H. N., Matta M. (2025). Atomistic polymer modeling: Recent
advances and challenges in building
and parametrization workflows. Macromolecules.

[27] Hopkins S. D., Blaisten-Barojas E. (2023). Molecular
dynamics simulations evidence the thermoresponsive
behavior of PNIPAM and PDEA in glycerol solutions. Front. Nanotechnol..

[28] Becke A. D. (1993). Density
functional thermochemistry. III. The role of exact exchange. J. Chem. Phys..

[29] Lee C., Yang W., Parr R. G. (1988). Development
of the Colle-Salvetti
correlation-energy formula into a functional of the electron density. Phys. Rev. B:Condens. Matter Mater. Phys..

[30] Antony J., Ehrlich S., Krieg H. (2010). A consistent and accurate ab initio
parameterization of density functional dispersion correction (DFT-D)
for the 94 elements H-Pu. J. Chem. Phys..

[31] Singh U. C., Kollman P. A. (1984). An approach to computing electrostatic charges for
molecules. J. Comput. Chem..

[32] Frisch, M. ; Trucks, G. W. ; Schlegel, H. B. ; Scuseria, G. E. ; Robb, M. A. ; Cheeseman, J. R. ; Gaussian 16 Revision C.01. 2016; Gaussian Inc.: Wallingford CT, 2013.

[33] Hopkins, S. D. ; Blaisten-Barojas, E. Molecular Dynamics Simulation of Thermoreactive Polymers: Open access topology files. 2025, 10.5281/zenodo.17517351.

[34] Lindahl, E. ; Abraham, M. ; Hess, B. ; van der Spoel, D. ; , GROMACS 2018.8: Documentation. 2018; https://manual.gromacs.org/documentation/2018-current/index.html.

[35] Nosé S. (1984). A molecular
dynamics method for simulations in the canonical ensemble. Mol. Phys..

[36] Nosé S., Klein M. (1983). Constant pressure molecular
dynamics for molecular systems. Mol. Phys..

[37] Parrinello M., Rahman A. (1981). Polymorphic transitions
in single crystals: A new molecular
dynamics method. J. Appl. Phys..

[38] Hess B., Bekker H., Berendsen H. J., Fraaije J. G. (1997). LINCS: A linear
constraint solver for molecular simulations. J. Comput. Chem..

[39] Essmann U., Perera L., Berkowitz M. L., Darden T., Lee H., Pedersen L. G. (1995). A smooth particle
mesh Ewald method. J. Chem. Phys..

[40] Herrera-Rodríguez A. M., Vedran M., Aponte-Santamaría C., Gräter F. (2019). Molecular
dynamics simulations of molecules in uniform flow. Biophys. J..

[41] Herrera-Rodríguez A. M., Dasanna A. K., Daday C., Cruz-Chú E. R., Aponte-Santamaría C., Schwarz U. S., Gräter F. (2023). The role of
flow in the self-assembly of dragline spider silk proteins. Biophys. J..

[42] Rumble, J. E. CRC Handbook of Chemistry and Physics; 98th ed.; CRC Press: Boca Raton, FL, 2017.

[43] Schröter K., Donth E. (2000). Viscosity
and shear response at the dynamic glass transition of glycerol. J. Chem. Phys..

