# Supplementary material for: Thermoresponsive Polymers under Solvent Flow through Molecular Dynamics
Source: J Phys Chem B. 2026 Mar 13;130(12):3484–93. doi: 10.1021/acs.jpcb.5c07833 (PMC13034415; doi:10.1021/acs.jpcb.5c07833)
Supplement: Supplementary file 1 [file jp5c07833_si_001.pdf]

Supporting Information
Thermoresponsive Polymers under Solvent Flow through Molecular Dynamics

Scott D. Hopkins and Estela Blaisten-Barojas*

Center for Simulation and Modeling (formerly, Computational Materials Science Center) and Department of Computational and Data Sciences, George Mason University, Fairfax, Virginia 22030, USA

E-mail: blaisten@gmu.edu

S1 Simulation parameters

The non-equilibrium molecular dynamics study pertains to systems containing 30-PNIPAM or 30-PDEA in solution with three distinct solvents, pure water, 50:50 w/w water:glycerol, and pure glycerol. A direct flow was applied in all simulations. A set of three simulations with slightly different initial conditions were performed. Note that 30-PNIPAM and 30-PDEA have 572 and 662 atoms, respectively. Parameters used in these simulations are summarized in Tables S1 and S2. The custom calculated atomic partial charges for these two polymer chains in their globule structure are part of the GROMACS topology files. The topology files are open access available at https://dx.doi.org/10.5281/zenodo.17517351.

Table S1: 30-PNIPAM molecular dynamics system configurations of the three simulations/system undertaken in this work. Energies are per atom.

solvent	trial	N_{water}	$N_{glycerol}$	C_{poly} (wt%)	T (K)	ρ (kg/m^3)	E_{tot} (kJ/mol)
water	1	16134	0	1.16	300	999 ± 2	-15.47 ± 0.02
					320	989 ± 2	-15.07 ± 0.02
	2	16141	0	1.15	300	999 ± 2	-15.47 ± 0.02
					320	988 ± 2	-15.07 ± 0.02
	3	16142	0	1.15	300	1000 ± 2	-15.47 ± 0.02
					320	988 ± 2	-15.07 ± 0.02
50:50	1	8257	1620	1.13	320	1119 ± 2	-10.47 ± 0.02
					340	1102 ± 2	-10.08 ± 0.02
	2	8252	1620	1.13	320	1120 ± 2	-10.47 ± 0.02
					340	1103 ± 2	-10.08 ± 0.02
	3	8261	1620	1.13	320	1118 ± 2	-10.47 ± 0.02
					340	1102 ± 2	-10.08 ± 0.02
glycerol	1	0	3440	1.06	380	1202 ± 3	-4.03 ± 0.03
					400	1184 ± 3	-3.66 ± 0.03
	2	0	3446	1.06	380	1203 ± 3	-4.03 ± 0.03
					400	1185 ± 3	-3.66 ± 0.03
	3	0	3441	1.06	380	1200 ± 3	-3.95 ± 0.03
					400	1182 ± 3	-3.58 ± 0.03

S2 Details on the direct flow simulations

The non-equilibrium MD pull-force method for generating a direct flow in the liquid contained in the computational box produces a slight gradient of the density along the length of the elongated computational box. To investigate this effect, 50 slices of the box were considered across the cross section perpendicular to the 20 nm elongated box dimension (x axis). The

Table S2: 30-PDEA molecular dynamics system configurations of the three simulations per system undertaken in this work. Energies are per atom.

solvent	trial	N_{water}	$N_{glycerol}$	C_{poly} (wt%)	T (K)	ρ (kg/m^3)	E_{tot} (kJ/mol)
water	1	16110	0	1.30	300	999 ± 2	-15.37 ± 0.02
					320	988 ± 2	-14.97 ± 0.02
	2	16091	0	1.30	300	998 ± 2	-15.37 ± 0.02
					320	988 ± 2	-14.97 ± 0.02
	3	16119	0	1.30	300	999 ± 2	-15.38 ± 0.02
					320	987 ± 2	-14.97 ± 0.02
50:50	1	1620	8238	1.27	340	1110 ± 2	-10.18 ± 0.02
					360	1093 ± 3	-9.79 ± 0.02
	2	1620	8279	1.26	340	1110 ± 2	10.19 ± 0.02
					360	1092 ± 3	9.80 ± 0.02
	3	1620	8265	1.26	340	1109 ± 2	-10.19 ± 0.02
					360	1091 ± 3	-9.80 ± 0.02
glycerol	1	0	3437	1.19	390	1191 ± 3	-3.76 ± 0.03
					410	1172 ± 3	-3.39 ± 0.03
	2	0	3419	1.20	390	1190 ± 3	3.76 ± 0.03
					410	1172 ± 3	3.40 ± 0.03
	3	0	3431	1.19	390	1191 ± 3	-3.77 ± 0.03
					410	1172 ± 3	-3.40 ± 0.03

liquid density in each computational box slice was calculated by averaging over 50 ns of flowing liquid. The system density profile is shown in Fig. S1, while the average density remains as reported in tables S1 and S2. The custom calculated partial atomic charges of the 30-PNIPAM and 30-PDEA are part of the topology files provided in the open access repository at https://dx.doi.org/10.5281/zenodo.17517351.

In the non-equilibrium MD simulations the polymer changes structure from the initial

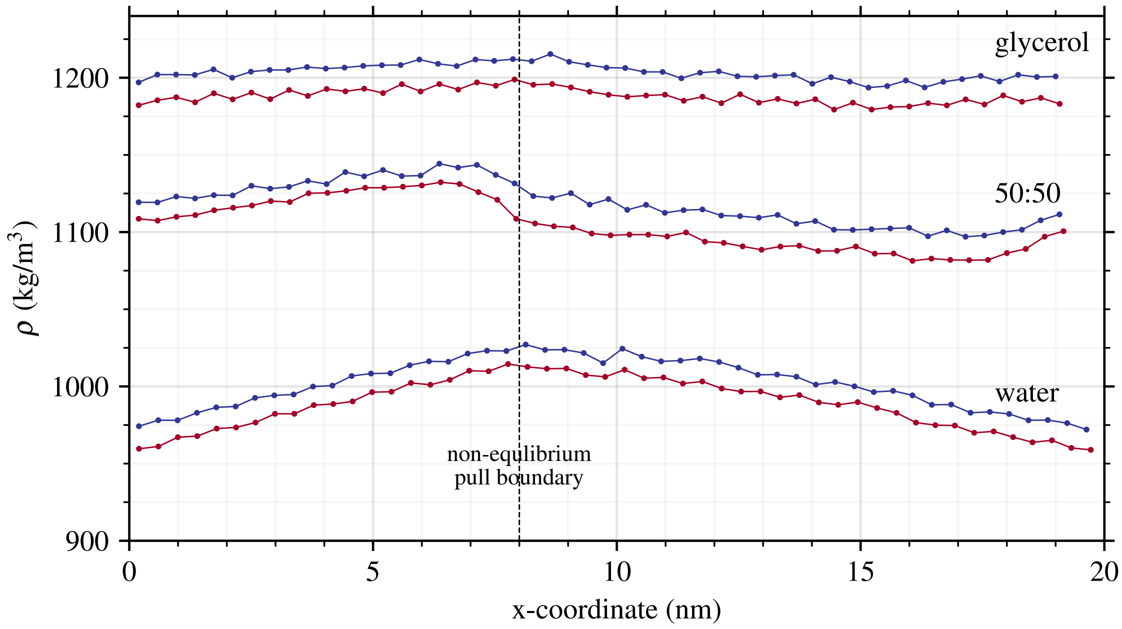

Figure S1: System density along the length of the computational box for 30-PNIPAM in flowing water, 50:50 w/w glycerol: water, and pure glycerol at the LCST (blue) and LCST+20 K (red). The location up to where the pull force was applied for obtaining the threshold flow velocity is depicted with a vertical doted line.

globular to an extended coil because of the established direct flow in the system. This structural change takes time to occur and depends upon the system temperature as well as on the flow velocity.

Figures S2 through S5 show the evolution along the last 30 ns of the 200 ns simulations of both, 30-PNIPAM and 30-PDEA, radius of gyration R_g, end-to-end distance R_{ee}, SASA (solvent accessible surface area), and the flow applied drag force F_d.

The radial distribution function (RDF) between the center of mass of each monomer in the polymer chain and the center of mass of each solvent molecule was calculated for the globule and extended coil structures of 30-PNIPAM and 30-PDEA in each of the three flowing solvents considered as shown in Fig. S6.

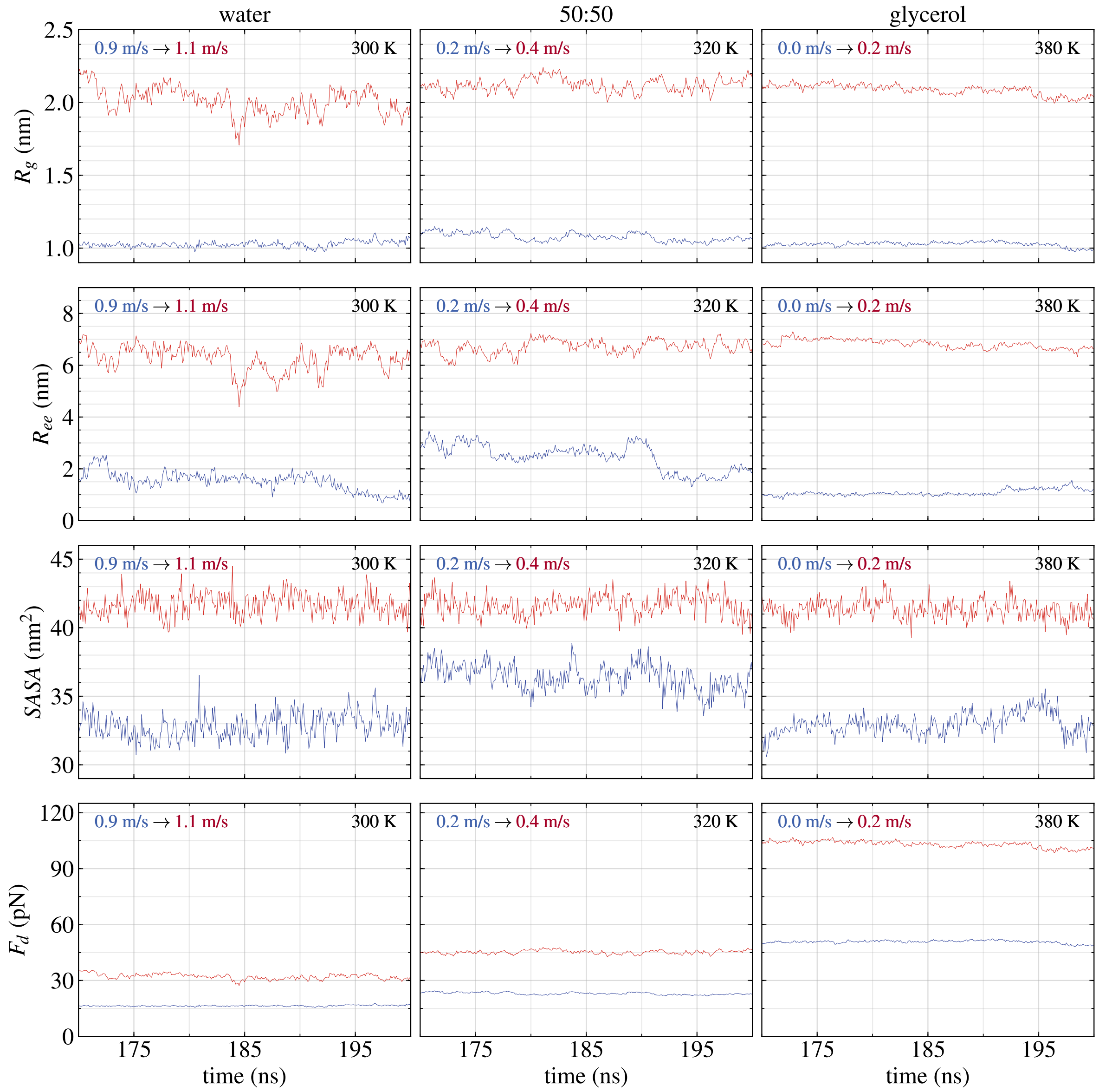

Figure S2: Time dependent structural properties of 30-PNIPAM at the LCST in water (300 K), 50:50 glycerol: water (320 K), and glycerol (380 K) at both, the flow threshold velocity (red) and below the flow threshold velocity (blue) of each solvent.

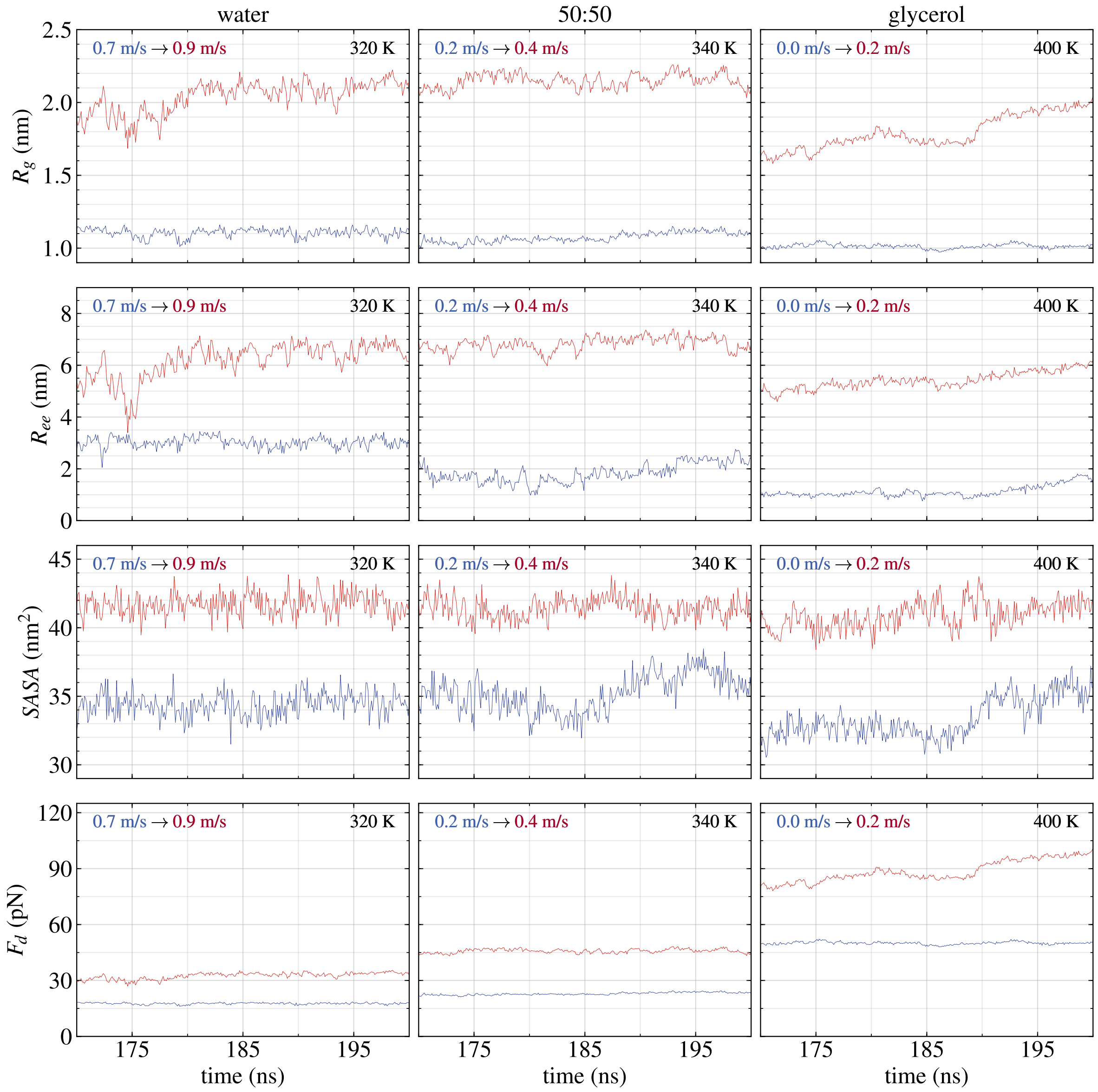

Figure S3: Time dependent structural properties of 30-PNIPAM at the LCST+20 K in water (320 K), 50:50 glycerol: water (340 K), and glycerol (400 K) at both, the flow threshold velocity (red) and below the flow threshold velocity (blue) of each solvent.

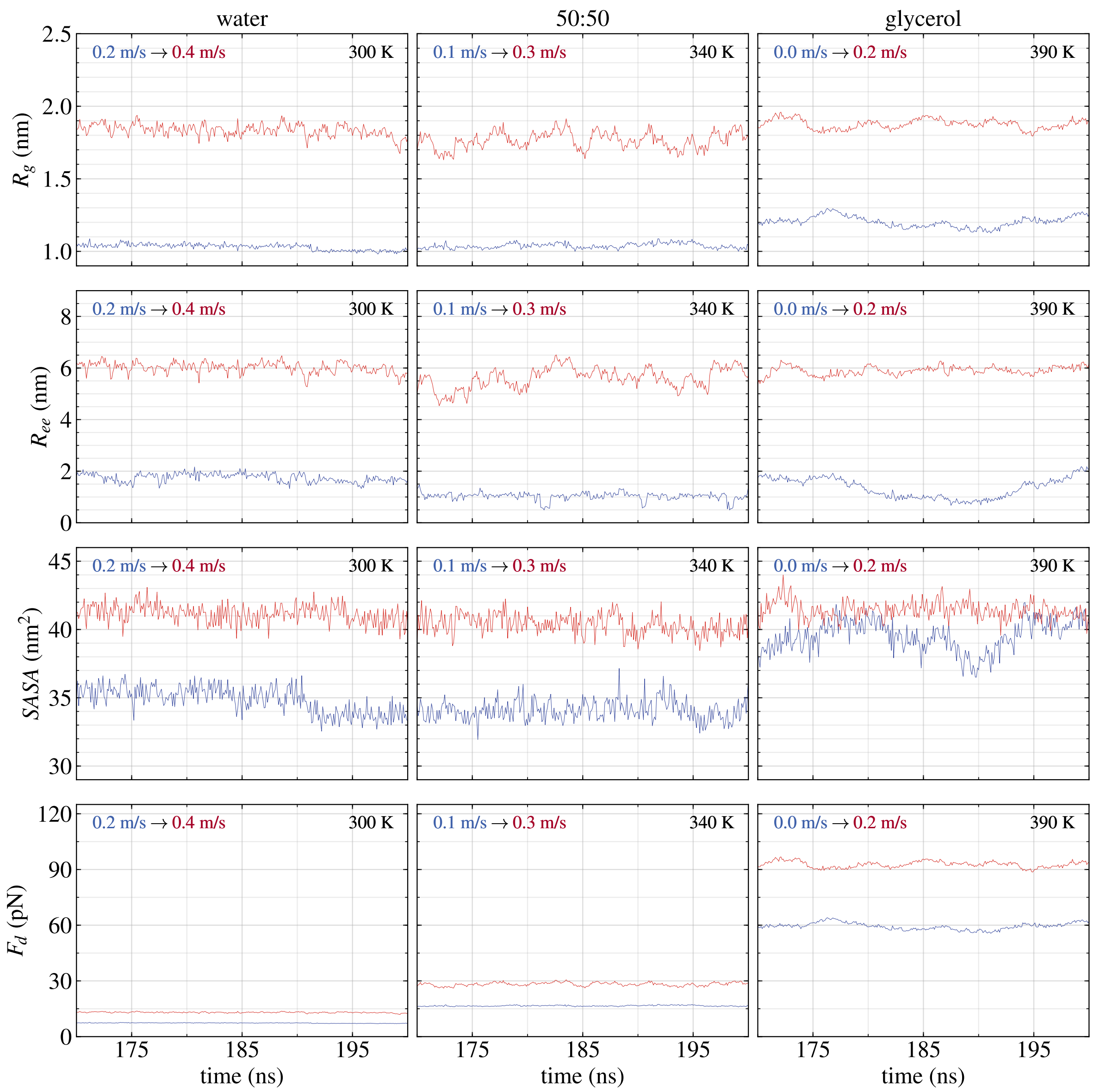

Figure S4: Time dependent structural properties of 30-PDEA at LCST in water (300 K), 50:50 glycerol: water (340 K), and glycerol (390 K) at, both, the flow threshold velocity (red) and below the flow threshold velocity (blue) of each solvent.

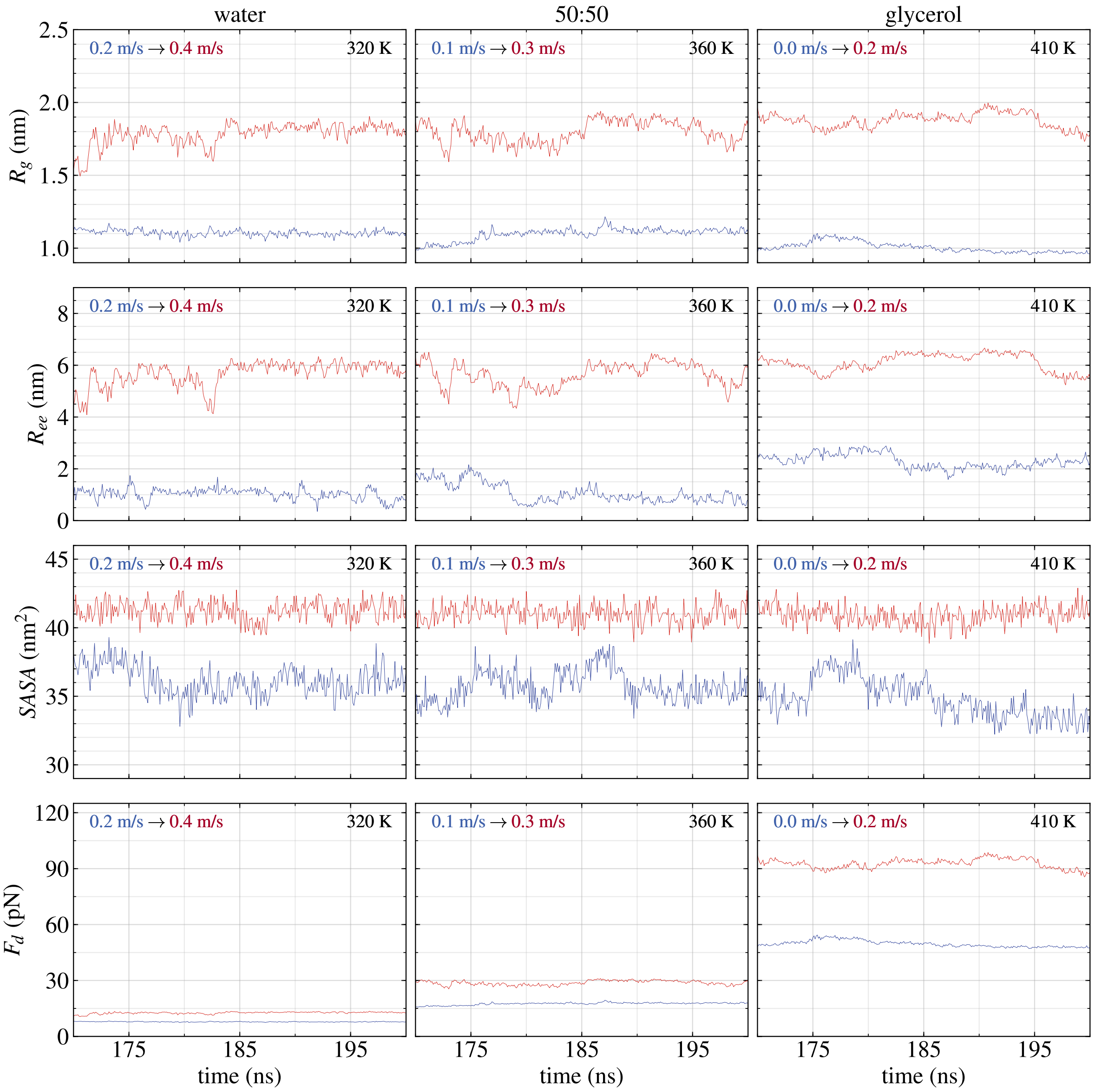

Figure S5: Time dependent structural properties of 30-PDEA at LCST+20 K in water (320 K), 50:50 glycerol:water (360 K), and glycerol (410 K) at both, the flow threshold velocity (red) and below the flow threshold velocity of (blue) of each solvent.

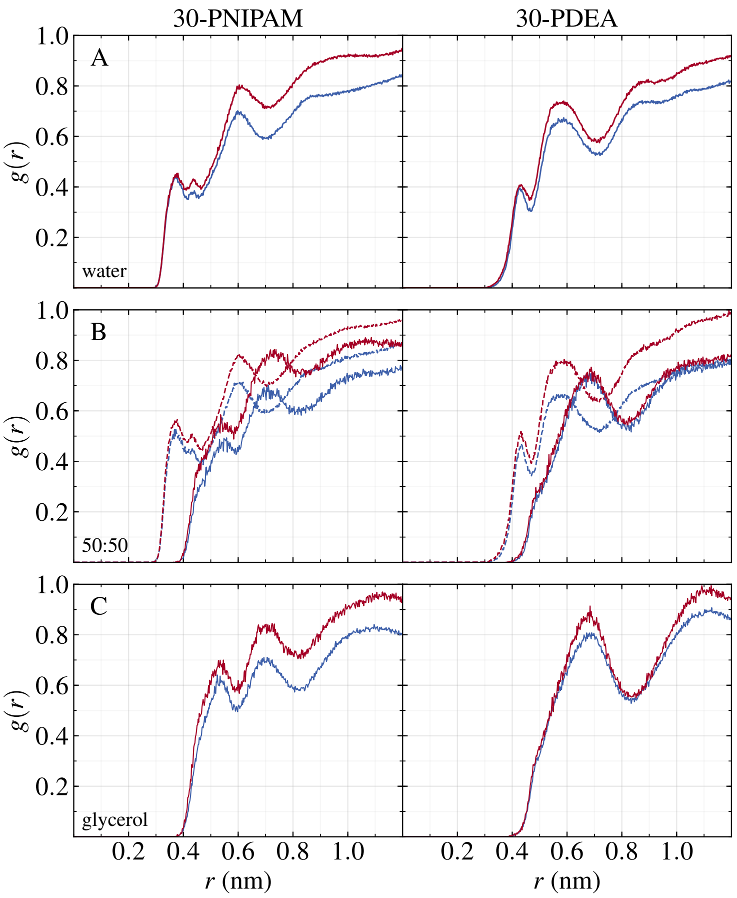

Figure S6: Radial distribution functions between the centers of mass of the solvent molecules and the polymer chain monomers in 30-PNIPAM (left) or 30-PDEA (right) in the globule (blue) and extended coil (red) structures. (A) water, (B) 50:50 glycerol:water mixture, (C) glycerol. For the 50:50 glycerol:water mixture, solid lines correspond to glycerol molecules, and dotted lines correspond to water molecules.
